# A High-Level Disinfection Standard for Land-Applied Sewage Sludges (Biosolids)

**DOI:** 10.1289/ehp.6207

**Published:** 2004-02

**Authors:** David K. Gattie, David L. Lewis

**Affiliations:** 1 Department of Biological and Agricultural Engineering, and; 2 Department of Marine Sciences, University of Georgia, Athens, Georgia, USA

## Abstract

associated with land-applied sewage sludges primarily involve irritation of the skin, mucous membranes, and the respiratory tract accompanied by opportunistic infections. Volatile emissions and organic dusts appear to be the main source of irritation. Occasionally, chronic gastrointestinal problems are reported by affected residents who have private wells. To prevent acute health effects, we recommend that the current system of classifying sludges based on indicator pathogen levels (Class A and Class B) be replaced with a single high-level disinfection standard and that methods used to treat sludges be improved to reduce levels of irritant chemicals, especially endotoxins. A national opinion survey of individuals impacted by or concerned about the safety of land-application practices indicated that most did not consider the practice inherently unsafe but that they lacked confidence in research supported by federal and state agencies


### A High-Level Disinfection Standard for Land-Applied Sewage Sludges (Biosolids)

***David K. Gattie[1] and David L. Lewis [2]***

[1]Department of Biological and Agricultural Engineering, and [2]Department of Marine Sciences, University of Georgia, Athens, Georgia, USA

**Complaints associated with land-applied sewage sludges primarily involve irritation of the skin, mucous membranes, and the respiratory tract accompanied by opportunistic infections. Volatile emissions and organic dusts appear to be the main source of irritation. Occasionally, chronic gastrointestinal problems are reported by affected residents who have private wells. To prevent acute health effects, we recommend that the current system of classifying sludges based on indicator pathogen levels (Class A and Class B) be replaced with a single high-level disinfection standard and that methods used to treat sludges be improved to reduce levels of irritant chemicals, especially endotoxins. A national opinion survey of individuals impacted by or concerned about the safety of land-application practices indicated that most did not consider the practice inherently unsafe but that they lacked confidence in research supported by federal and state agencies. *Key words*: biosolids, sewage sludge. *Environ Health Perspect* 112:126–131 (2004). doi:10.1289/ehp.6207 available via *http://dx.doi.org/* [Online 17 November 2003]**

Applying processed sewage sludges (biosolids) to crop land, strip mines, public parks, and other areas has become common in the United States and elsewhere. This practice, in which several tons or more of processed municipal wastes are applied per acre annually, is regulated by the U.S. Environmental Protection Agency (EPA) under the 503 sludge rule (U.S. EPA 1993). The rule provides guidance for the beneficial reuse of municipal wastes and sets standards intended to protect public health and the environment from exposure to heavy metals, toxic chemicals, and pathogens. In recent years, land application has been increasingly scrutinized because of nuisance complaints and growing numbers of anecdotal reports of illnesses and deaths attributed to exposure to commercially processed sewage sludges.

Our laboratories investigated public complaints and concluded that irritant chemicals associated with volatile chemicals and dusts blowing from treated land (e.g., bacterial toxins, lime, organic amines) may cause nearby residents to be more susceptible to infections (Lewis et al. 2002, Lewis and Gattie 2002). We documented an outbreak of *Staphylococcus aureus* among individuals exposed to a Pennsylvania land-application site and attributed the infections to secondary exposure routes (animal-to-human or person-to-person). Overall, we questioned the efficacy of methods used to treat sewage sludges and determine pathogen levels; and, we recommended that new research focus on chemical–pathogen interactions, airborne contaminants (especially organic dusts), and risks posed to immunocompromised individuals and other sensitive populations (Lewis 1998; Lewis et al. 1999, 2000, 2001, 2002; Lewis and Gattie 2002).

The National Research Council (NRC 2002) echoed these same concerns, and the U.S. EPA intends to address some of the issues through additional research (U.S. EPA 2003a). In this paper we provide a more detailed overview of the risks that land application of sewage sludge poses to human health and how those risks can be better managed.

### High-Level Disinfection

Current federal standards for pathogen reduction in sewage sludge are based on levels of indicator organisms, such as *Escherichia coli* and *Salmonella*. Class A sludges have no detectible pathogens, whereas low levels of indicator pathogens are permitted in class B sludges. Sludges contain a wide variety of bacteria, viruses, protozoa, fungi, and parasitic worms, including some species that are more difficult to kill than the indicator organisms.

Table 1 shows levels of disinfection required to destroy different groups of pathogens found in sewage sludges. Low-level disinfection reduces numbers of vegetative bacteria (e.g., *E. coli*, *Salmonella*) and enveloped viruses [e.g., hepatitis B, human immunodeficiency virus (HIV), influenza viruses]. More resistant organisms require intermediate-level disinfection. These include mycobacteria (e.g., *Mycobacterium tuberculosis*), protozoa (e.g., *Cryptosporidium*, *Giardia*), parasitic worms (e.g., *Ascaris*, *Toxocara*) and fungi (e.g., *Candida*). Intermediate to high-level disinfection is required to kill some of the most important pathogens found in sludges, including small, nonenveloped viruses (e.g., *Norovirus*) and bacterial endospores (e.g., *Clostridium perfringens*).

Nonenveloped viruses comprise an important group of pathogens that require a higher level of disinfection than the indicator organisms recommended in the 503 rule (U.S. EPA 1993). Rotaviruses, for example, cause 30–40% of acute diarrhea that requires infants to be hospitalized, and *Norovirus* (Norwalk-like viruses) is responsible for 40% of the cases of nonbacterial diarrhea in children and adults (Berkow and Fletcher 1992). Other important infectious agents in this group include hepatitis A, hepatitis E, encephalomyocarditis virus, polioviruses, coxsacki viruses, reoviruses, rhinoviruses, astroviruses, caliciviruses, echoviruses, parvoviruses, and aphthovirus. Many of these viruses pose a particular threat to infants, the elderly, and individuals with chronic diseases.

The National Institute for Occupational Safety and Health (NIOSH 2002) recently concluded that Class B biosolids likely contain infectious levels of bacteria, viruses, protozoa, and helminths and recommended that workers use protective gear and take basic infection control precautions when handling the material. In issuing these guidelines, NIOSH acknowledged that current methods for processing Class B sewage sludges may fail to achieve even low-level disinfection.

Also recognizing that freshly processed Class B sludges may pose a significant risk of infection under certain conditions, the U.S. EPA included protective measures in the 503 rule (U.S. EPA 1993), such as temporarily restricting public access to Class B land-application sites with warning signs and fences. The U.S. EPA, however, failed to consider some potentially important exposure factors; for example, dusts from treated fields could expose surrounding communities, and certain chemicals in sludge may increase risks of infections. Moreover, stockpiling sludge and spreading it without incorporating it into soil are commonplace. In practice, the 503 rule is ineffective in preventing public exposure.

Based on the types of pathogens present in municipal wastes, sewage sludges should be treated with high-level disinfection. To meet this standard, treatment methods should demonstrate the ability to kill even the most

Address correspondence to D.K. Gattie, Department of Biological and Agricultural Engineering, Driftmier Engineering Center, University of Georgia, Athens, GA 30602-4435 USA. Telephone: (706) 542-0880. Fax: (706) 542-8806. E-mail: dgattie@engr.uga.edu

We thank C. Snyder, Sierra Club Sludge Task Force, for her assistance in taking a national survey of public concerns. We also thank M. Novak for assisting with the survey and providing other technical support.

The authors declare they have no competing financial interests.

Received 13 January 2003; accepted 17 November 2003.

resistant organisms, including nonenveloped viruses and bacterial spores. Because all federal and state requirements are based on less-resistant indicator organisms, it is not known whether current methods, including aerobic and anaerobic digestion, heat treatment, lime stabilization, and composting, could achieve high-level disinfection.

### Pathogen Regrowth

Although high-level disinfection would afford greater protection for both workers and the public from pathogens in freshly processed sewage sludge, the public can also be exposed to pathogens that proliferate after the sludge is applied (Gibbs et al. 1997). Viruses do not replicate outside their hosts; therefore, pathogen regrowth is mainly of concern with bacteria and fungi. Consequently, while viruses and other pathogens die off in the field, some pathogens may rebound. Also, new pathogens are introduced when sludge is mixed with soil and comes in contact with insects, birds, mammals, and other environmental sources of pathogens.

The potential for pathogen regrowth is the downside to sewage sludge being rich in nutrients that promote the growth of bacteria and fungi. The problem is similar to food poisoning with perishable foods, such as egg products. Eggs, like raw sewage, are often contaminated with *Salmonella*. With a little cooking, however, egg-containing products are safe for human consumption. Nevertheless, unless these foods are desiccated or refrigerated, other pathogens, such as *S. aureus,* multiply in them. The source of *S. aureus* in spoiled food is not the eggs, however, but normal skin microflora from the hands of people who prepare or handle the food.

Although sewage sludge is not a food product, the principle is the same. Sludge is rich in proteins and other nitrogen-rich organic compounds that promote the growth of *S. aureus* and other bacteria. These organisms multiply as sludges decompose in soil, and can present a risk of infection when traces of sludge enter skin abrasions or when the dusts contact mucous membranes or are inhaled. The risk is particularly high when sewage sludge contacts tissues injured by chemical irritants, burns, cuts, or abrasions. People with chronic diseases and compromised immune systems are especially at risk.

Also, as is the case with food products, sewage sludge that is heated or otherwise treated to kill pathogens is still subject to pathogen regrowth. In fact, because most of the competing microorganisms are eliminated, it is even more conducive to pathogen regrowth. Leaving pathogens in sewage sludge, however, is not the solution.

Unfortunately, pathogen regrowth is an inherent problem with all sludges rich in proteins, amino acids, and other forms of organic nitrogen and sulfur—regardless of how they are processed. Once the materials are applied and become wet, they are colonized by bacteria and fungi; the materials then decompose and emit noxious odors in the form of organic amines, organic sulfides, and other small-molecular-weight compounds.

Offensive odors that form as sludge biologically decomposes in the field indicate pathogen regrowth because they are produced as bacteria break down proteins and other organic compounds containing nitrogen and sulfur. Most treatment methods produce sludges that are only temporarily stable; that is, the sludges produce noxious odors from biological decomposition after they are applied in the field.

One commercial process achieves long-term stability by chemically reacting sludge under heat and pressure at high pH to drive off organic nitrogen as ammonia (Reimers et al. 2003). With this process, the combination of gaseous ammonia, high temperature, and pressure effectively eliminates a wide range of pathogens. The final wet product, which is odorless and has a high pH, is used to amend acidic soils. Because the nitrogen content is driven off, however, the product lacks nutrient value.

### Bacterial Toxins

Most bacteria found in sewage sludge produce either endotoxins or exotoxins, both of which can cause severe illness or death. As sludges decompose, toxins can leach into groundwater, enter surface water runoff, and be carried away in airborne dusts. Considering that tons of decomposing sewage sludge per acre are often applied to hundreds or thousands of acres many times a year, land-application sites have a potential for producing and exporting large quantities of toxins.

Exotoxins—proteins and peptides secreted into the surrounding environment by growing cells—are produced by both gram-negative and gram-positive bacteria. They are usually the most toxic of the two general types of bacterial toxins. Because they can retain their toxicity at extremely high dilutions, some exotoxins, including staphylococcal enterotoxins and shigatoxin, are used as biological warfare agents.

Although exotoxins are generally heat labile and could therefore be destroyed by heat-treatment processes for sewage sludges, treated sludges are still likely to become contaminated with *E. coli*, *Pseudomonas auruginosa,* and other exotoxin-producing bacteria in the field. Severe gastrointestinal illnesses reported by individuals using private wells near land-application sites may have been caused by exotoxins leaching into groundwater.

The same property that makes *S. aureus* a common cause of food poisoning—its ubiquitous presence—may also make it one of the more common pathogens to proliferate in sewage sludges after they are applied to land. The organism produces an exotoxin that is not destroyed by cooking. Symptoms caused by *S. aureus* food poisoning (e.g., nausea, cramps, vomiting) are due to the presence of this toxin. Land-application sites with high levels of *S. aureus* could contaminate air and water with potentially harmful levels of both the organism and its toxin.

Endotoxins, on the other hand, are lipopolysaccharide complexes in the cell walls of gram-negative bacteria only. They are associated with proteins and other components of the cell walls and are released when the bacteria die and cell walls break apart (Rylander 1995). Endotoxins are produced in large quantities when wastes colonized with gram-negative bacteria are treated (Sigsgaard et al. 1994). They would also be produced as gram-negative bacteria growing in nutrient-rich sludges die off in the field.

Unlike most exotoxins, endotoxins are heat stable even upon autoclaving (Baines 2000). They can, however, be inactivated with dry heat at > 200°C for 1 hr (Williams 2001). Traces of endotoxins in food and water can cause headaches, fever, fatigue, and severe gastrointestinal symptoms; however, their primary target is the lungs. In addition to the former symptoms, inhaling endotoxin-contaminated dusts can cause acute airflow obstruction, shock, and even death. Chronic respiratory effects can also develop [American Conference of Government Industrial Hygienists (ACGIH) 1999].

**Table 1.** Disinfection levels required to kill pathogens in sewage sludges.[a]

| Group | Disinfection level required |
|---|---|
| Bacterial endospores (e.g., *Bacillus anthracis*) | High |
| Nonenveloped viruses (e.g., *Norovirus*, *Coxsackie*, *Rotavirus*) | Intermediate/high |
| Helminths (e.g., *Ascaris*, *Toxocara*) | Intermediate |
| Protozoa (e.g., *Cryptosporidium*, *Giardia*) | Intermediate |
| Mycobacteria (e.g., *M. tuberculosis*) | Intermediate |
| Fungi (e.g., *Candida*) | Low/intermediate |
| Vegetative bacteria (e.g., *Staphylococcus*, *Salmonella*) | Low |
| Enveloped viruses (e.g., hepatitis B, HIV, influenza) | Low |

Data from the Association for the Advancement of Medical Instrumentation (AAMI 1994).
[a]Disinfection levels are based on susceptibilities to liquid chemical germicides; groups increase similarly in resistance to heat, with enveloped viruses being the most sensitive and bacterial endospores the most resistant.

Allergic and nonallergic reactions caused by airborne endotoxins have been documented with exposures of 45–150 endotoxin units (EU)/$m^3$ and 300–400 EU/$m^3$ (Milton et al. 1996; Smid et al. 1994). Nearby residents exposed to dusts from land-application sites report many of the same symptoms of endotoxin poisoning that have been documented among sewage treatment plant workers. These include flu-like symptoms, nausea, vomiting, diarrhea, headaches, and difficulty breathing (Lewis et al. 2002). Rylander (1987) proposed occupational exposure limits to endotoxin-contaminated cotton dusts. Based on average air concentrations over an 8- to 10-hr workday, he suggested limits ranging from 200 EU/$m^3$ to prevent airway inflammation to 20,000 EU/$m^3$ to avoid toxic pneumonitis. The exposure levels of endotoxin-contaminated aerosols with sewage treatment plant workers have ranged from 80 to 4,100 EU/$m^3$ (Liesvuori et al. 1994). The toxins, however, have a greater effect on people with immune systems compromised by injury or illness (Baines 2000).

### Chemical–Pathogen Interactions

Although many chemical contaminants found in processed sewage sludges may potentially interact with pathogens to cause, facilitate, or exacerbate the disease process through allegeric and nonallergic mechanisms, microbial byproducts formed during the processing and decomposition of sewage sludge probably account for most of the acute health effects. Complaints among residents living near land-application sites are primarily respiratory related and are consistent with hypersensitivity reactions, including fever, cough, difficulty in breathing, nausea, and vomiting.

Numerous diseases involving immunologically mediated hypersensitivity reactions have been documented among workers exposed to organic dusts containing microbial products. Yi (2002) listed 27 diseases, each categorized according to the source of the dusts and the specific microorganisms identified as the primary cause of hypersensitivity. Sources include, for example, dusts from molded hay, mushroom compost contaminated with fungi and actinomycetes, *Streptomyces*-contaminated fertilizers, *Caphaloporium*-contaminated sewage, and wood contaminated with *Bacillus subtilis*.

Byssinosis, perhaps the most studied of these diseases, is attributed to traces of endotoxins from the breakdown of *E. coli* and other gram-negative bacteria on raw cotton fibers. Similarly, illnesses have been documented among wastewater treatment plant workers exposed to endotoxins in aerosols (Rylander 1987). Usually, the disease affects only a small percentage of sensitive workers.

Compared with waste treatment plant aerosols, however, endotoxin levels are probably much higher in sewage sludge dusts, which contain large numbers of predominantly gram-negative bacteria killed during treatment processes and after land application. Consequently, the frequency and severity of hypersensitivity among groups exposed to sewage sludge dusts may be much greater compared with exposure to other organic dusts.

Respiratory-related hypersensitivity is generally reversible when affected individuals are removed from the source of exposure and treated with high doses of corticosteroids. Corticosteroids used to treat the underlying inflammation, however, seriously impair the immune system. In the case of sewage sludge, this would render hypersensitive individuals highly susceptible to infection from the low levels of viruses, bacteria, fungi, and other pathogens in processed sludges.

Treating residents near land-application sites who experience hypersensitivity to processed sewage sludges, therefore, is both costly and risky. It would involve relocating affected individuals to another area, making certain that any potentially serious infections have been eliminated or controlled with antibiotics, then administering high doses of corticosteroids and closely monitoring for any new infections.

### Exposure Studies

A conference on health effects of odors sponsored by Duke University and the U.S. EPA concluded that gases and volatile emissions from waste products including processed sewage sludge may cause adverse health effects (Schiffman et al. 2000). While acknowledging the complexity of the problem, the participants recommended undertaking controlled studies of the odorous emissions. In responding to the NRC recommendations (NRC 2002), the U.S. EPA committed to measuring field concentrations of selected volatile and gaseous compounds at selected sites (U.S. EPA 2003a).

Land-applied sewage sludge can emit numerous volatile chemicals and gases that may act alone or in combination with one another to produce the kinds of symptoms reported by people living near biosolids-recycling operations. The composition of air contaminants emitted by any land-application site undoubtedly varies widely over space and time, as do the susceptibilities of individuals to the effects of these emissions. Consequently, it is unlikely that such research will adequately establish which components and combinations of components can potentially cause adverse health effects and under what conditions.

We propose an alternative approach with a more modest goal aimed at determining the extent to which emissions must be diluted to eliminate malodor complaints and irritant effects (e.g., burning eyes, coughing, breathing difficulties). Based on meteorologic data from local weather stations and publicly available topographic data, the dilution of air contaminants over areas surrounding land-application sites can be readily determined with an air-dispersion model (Lewis et al. 2002).

Meteorologic data should be collected over an extended period of time (e.g., 6–8 weeks) during the maximum potential exposure, for example, when land application is in progress, temperatures are high, and sufficient rainfall has occurred to support high levels of microbial activity. This approach does not require measuring specific pollutants, and a number of land-application sites could be studied with a reasonable level of resources.

In addition to collecting meteorologic data, local census data would be used in this type of study to randomly select two groups of residents of similar demographic compositions: one close to the land-application site (< 1 km) and one farther away (3–5 km). Individuals in each group would provide information on medical histories and keep daily records of the selected symptoms during the period when meteorologic data are collected. Using a similar approach, we found that residents living closer to land-application sites were more severely affected than those living farther away (Lewis et al. 2002).

Quantitatively, what is needed is a simple numerical index that captures the most important variables determining whether symptoms develop. The amount by which volatile emissions are diluted when they reach a residence and the number of times the dilution drops below a certain level may be sufficient for predicting whether odor and health-related complaints are likely to develop.

For example, an exposure index could be calculated based on *a*) the number of exposures in which levels of volatile chemicals at a residence are ≥ 10% of the levels over the sludged field and *b*) the average percent dilution for these exposures. This approach is illustrated by Equation 1, where $I_v$ is the exposure index for volatile emissions, $n$ is the number of exposures in which levels of gases and volatile chemicals at a residence were ≥ 10% of the levels over the sludged field, and đ is the average dilution (percent) for all exposures ≥ 10% of the levels over the field.

$$I_v = n \cdot đ \quad [1]$$

Once exposure indices and frequencies of symptoms are collected for a number of land-application sites, a representative dilution level required to eliminate odor complaints and acute adverse health effects can be determined. This index provides a quantitative measure of whether a land-application site is likely to cause odor complaints and acute adverse health effects at a particular location or distance from the site. Such information could be used to evaluate the effectiveness of treatment methods and land management practices.

### Public Concerns

To assess public concerns over the safety of current land-application practices, we distributed questionnaires to 150 individuals concerned about land-application of sewage sludges (Table 2). The group included farmers, residents complaining of adverse health effects, community leaders, and environmentalists. Based on the responses of 87 respondents from 15 states, a majority of respondents (51.7%) desired a total ban on land application of sewage sludges, while 35.6% believed that land application should just be suspended until proven safe. Most respondents (74.7%) lived near land-applications sites and most (67.5%) reported that they had been personally affected by the practice. Overwhelming malodor, vector attraction (flies, mosquitoes), and adverse health effects (e.g., difficulty breathing, chronic sinusitis) were the primary adverse effects reported by individuals living near the sites.

The need for additional research was strongly supported. Respondents, however, expressed little trust in federal and state environmental agencies to provide a reliable scientific evaluation of potential public health and environmental effects. More confidence was expressed if assessments were done by public health agencies, such as the Centers for Disease Control and Prevention (CDC) and

**Table 2.** Summary of survey results from 87 respondents indicating their level of public concern about land application practices.

| Topic | Question | Choices | Percent or mean ± SD (*n*) |
|---|---|---|---|
| Background information[a] | Why are you interested in the issue of land-applied sewage sludges? | Live or lived near land application site | 74.7% (87) |
| | | Work as a farmer/grower | 4.6% (87) |
| | | Engaged in environmental activism | 16.1% (87) |
| | | Other | 14.9% (87) |
| | Have you ever been personally affected by land application of sewage sludges? | Yes | 67.5% (77) |
| | How do you think land application of sewage sludges should be handled? | Current practices are safe;no new restrictions are needed | 0% (87) |
| | | All land application should be completely banned | 51.7% (87) |
| | | Only certain land application practices should be banned | 8.0% (87) |
| | | All land application should be suspended until proven safe | 35.6% (87) |
| | | Land application should be continued with certain new restrictions | 2.3% (87) |
| | | Other | 5.7% (87) |
| Level of concern[b] | On a scale of 0 to 10 (0 = no concern; 10 = highest level of concern), indicate your level of concern regarding the following issues | Microorganisms that may cause infection | 9.7 ± 0.9 (83) |
| | | Chemicals, metals and microorganism that may cause cancer | 9.6 ± 1.0 (84) |
| | | Odor-causing emissions | 8.9 ± 1.9 (84) |
| | | Bacterial toxins | 9.7 ± 0.8 (82) |
| | | Property value | 8.6 ± 2.3 (82) |
| | | Other | 9.7 ± 0.7 (25) |
| | Kinds of contamination from sludges that cause the most concern (0 = no concern; 10 = highest level of concern) | Contamination of food supply | 9.3 ± 1.4 (83) |
| | | Contamination of water | 9.9 ± 0.7 (84) |
| | | Contamination of soil | 9.8 ± 0.5 (84) |
| | | Contamination of air | 9.6 ± 1.0 (84) |
| | | Other | 9.8 ± 0.5 (26) |
| Level of trust[b] | Using a scale of 0 to 10 (0 = no trust; 10 = highest level of trust) indicate your level of trust in organizations dealing with land application of sewage sludges | Congress | 2.0 ± 2.3 (81) |
| | | U.S. EPA | 1.3 ± 2.4 (82) |
| | | U.S. Department of Agriculture | 1.7 ± 2.5 (80) |
| | | State agencies | 1.2 ± 2.1 (81) |
| | | Local governments (city/county) | 2.4 ± 3.1 (81) |
| | | Environmental organizations | 7.0 ± 2.8 (81) |
| | | Trade groups (e.g., WEF, NEBRA) | 0.8 ± 2.1 (75) |
| | | National Biosolids Partnership | 0.5 ± 1.7 (72) |
| | | Industry | 0.5 ± 1.5 (80) |
| | | Other[c] (e.g., departments of health, independent scientists) | 5.3 ± 4.4 (19) |
| Need for additional research[b] | On a scale of 0 to 10 (0 = don't feel that more research is needed; 10 = feel very strongly that more research is needed), indicate how strongly you feel that more scientific research is needed before we will know whether land applying sewage sludges is safe for public health and the environment | | 9.6 ± 1.7 (84) |
| | On a scale of 0 to 10 (0 = no trust; 10 = highest level of trust), indicate your level of trust in the work of scientists supported by various organizations dealing with land application of sewage sludges | National Science Foundation/National Institutes of Health | 5.1 ± 3.3 (72) |
| | | Trade groups (WEF/WERF) | 1.4 ± 2.2 (72) |
| | | U.S. EPA Office of Water | 1.4 ± 2.4 (77) |
| | | U.S. EPA Office of Research and Development | 2.8 ± 3.1 (75) |
| | | U.S. Department of Agriculture | 1.8 ± 2.6 (76) |
| | | Centers for Disease Control and Prevention | 4.9 ± 3.4 (75) |
| | | Industry | 0.5 ± 1.4 (76) |
| | | State agencies | 1.2 ± 2.1 (78) |
| | | Other[c] (e.g., universities, independent scientists, environmental groups) | 5.9 ± 4.7 (19) |

Abbreviations: NEBRA, New England Biosolids and Residuals Association; WEF, Water Environment Federation; WERF, Water Environment Research Federation. Responses not following survey instructions were omitted.
[a]Based on yes/no responses from all 87 respondents; values shown are percent ± SD (*n*). [b]Based on a 0–10 scale, with averages determined from the actual number of responses to each category; values shown are mean ± SD (*n*). [c]Categories given by respondents.

the National Institutes of Health (NIH). Although few respondents (16.1%) reported engaging in environmental activism, the group rated environmentalist organizations as being the most trustworthy to assess the safety of land-application practices.

Overall, the survey results indicate that most people concerned about sewage sludge do not believe land application is inherently unsafe but object to the practice because they lack confidence in scientific studies funded by government and industry groups defending the status quo. By contrast, survey respondents indicated a greater level of confidence in studies of land-application practices if done by the CDC or the NIH. It appears, therefore, that overcoming opposition through additional scientific research will require strong involvement with respected public health organizations. It will also require supportive findings from researchers independent of the federal agencies and trade organizations that have historically overseen the development and marketing of land-application practices.

### Discussion

***Politics of land application.*** Since the 503 sludge rule (U.S. EPA 1993) was promulgated in 1993, the U.S. EPA, the U.S. Department of Agriculture, and the industry and its trade associations have vigorously defended the rule as fully protective of public health and the environment. The primary basis has been a lack of documented cases of illnesses and the results of research supported with congressional funds earmarked for promoting land application as safe and beneficial (U.S. EPA 2002).

In 2000, the Committee on Science in the U.S. House of Representatives held hearings into allegations that the U.S. EPA retaliates against scientists and private citizens who report adverse environmental and health effects associated with sewage sludge (U.S. House of Representatives 2000a, 2000b).

During the hearings, the U.S. EPA Office of the Inspector General released a report confirming that concerns were widespread among U.S. EPA scientists who reviewed the 503 rule (U.S. EPA 2002). The assistant administrator refused to approve the rule without a major commitment from the Office of Water to support additional in-house research within the Office of Research and Development. The Inspector General noted that this research was never carried out.

The U.S. EPA responded to the congressional hearings by calling for a study by the National Research Council; Congress debated, and overwhelmingly passed, the No Fear Act (Notification and Federal Employee Antidiscrimination and Retaliation Act of 2002). The act, which was aimed at better protecting employees against retaliation, was signed by President Bush last year. The NRC published its findings and recommendations in July 2002 (NRC 2002), and the U.S. EPA addressed them in a research strategy in the *Federal Register* earlier this year (U.S. EPA 2003a). The U.S. EPA's final response to the NRC report is due to be released in January 2004. In the meantime, the U.S. EPA Office of Water has provided a docket for public comments (U.S. EPA 2003b).

Public comments to the Office of Water docket have largely mirrored those in the survey we report in this article. There is an overall lack of confidence in the the U.S. EPA's willingness to conduct or support objective research in this area. As evidence, the Sierra Club (San Francisco, CA) and others pointed to the fact that the Office of Water intends to address the NRC recommendations extramurally by funding the same researchers it has historically supported with congressional appropriations for promoting the safety of biosolids. The NRC study (NRC 2002), therefore, had its beginning and ending rooted in controversy over the U.S. EPA using congressional appropriations to support federal policies on the beneficial reuse of sewage sludge and to oppose scientists and private citizens who question them.

***Trends in land application.*** With increasing numbers of residents who live near sludged fields reporting respiratory and gastrointestinal illnesses (Shields 2003), many local governments have banned land application of Class B sewage sludges. However, we found that some Class A sludges generate the same complaints and concluded that going to Class A products will not resolve the pathogens issue (Lewis and Gattie 2002). The reason for this is that infections appear to be primarily opportunistic, following irritation of the skin, mucous membranes, and respiratory tract by chemical components.

Consequently, an important aspect of preventing infections lies in reducing levels of microbial toxins and other chemicals that cause inflammation as well as other responses that predispose individuals to infection. As such, the infections arise from many sources, both community and environmental. The problem with Class A sludges is probably primarily endotoxin related. This is because gram-negative bacteria comprise much of the biomass and because most conditions used to kill bacteria in treatment processes are insufficient to break down endotoxins. The Class A standard, therefore, while reducing the risks of acquiring infections directly from processed sludge, could increase risks of infections from other environmental and community sources.

One outcome of local bans is that land application of sewage sludge is being forced out of areas where residents have the political and economic resources to oppose the practice and into economically depressed areas. Whether this is intentional or not, sewage sludge is being dumped more and more into those communities least able to have their complaints heard, and where residents are least capable of relocating or obtaining medical treatment.

The changing demographics of land application of sewage sludge, therefore, need to be studied. First, census data should be used to assess the socioeconomic makeup of communities living near land-application sites (< 1 km away). Steps then need to be taken to ensure that land-application practices do not disproportionately impact low-income and minority communities.

### Recommendations

We recommend that the U.S. EPA undertake and complete five top-priority measures by January 2006 to address the immediate adverse health and environmental effects associated with land-applied sewage sludges:

- Develop a universal high-level disinfection standard to replace the current Class A/Class B standards, and require industry to provide efficacy data showing treatment methods meet this standard for sporocidal, fungicidal, bactericidal, tuberculocidal, virucidal, anti-protozoal, and anti-parasitic activity
- Develop treatment methods and land management practices for reducing airborne endotoxin levels associated with processed sewage sludges and set limits for public exposure. (We recommend that maximum levels be set at 0.1 times the limit recommended for 10-hr occupational exposure for preventing airway inflammation, which would be 20 EU/$m^3$.)
- Conduct a national assessment of groundwater contamination from pathogens, microbial (bacterial, fungal) toxins, organic chemicals, and metals at land-application sites
- Require that industry ensure that land-application practices do not disproportionately target low-income and minority subpopulations in rural communities
- Work with the National Biosolids Partnership (Association of Metropolitan Sewerage Agencies, U.S. EPA, and the Water Environment Federation) to develop and enforce a policy supporting open competition for research funding and prohibiting discrimination and retaliation against individuals raising concerns over adverse environmental and health effects from land-applied sewage sludges.

### References

ACGIH. 1999. Bioaerosols Assessment and Controls. Cincinnati, OH:American Conference of Government Industrial Hygienists.

AAMI. 1994. Designing, Testing & Labeling Reusable Medical Devices for Reprocessing in Health Care Facilities: A Guide for Device Manufacturers. Technical Information Report No. 12. Arlington, VA:Association for the Advancement of Medical Instrumentation.
Baines A. 2000. Endotoxin testing. In: Handbook of Microbiological Quality Control (Baird R, Hodges N, Denyer S, eds). New York:Taylor & Francis, 144–167.
Berkow R, Fletcher A. 1992. Gastroenteritis: infective and toxic. In: The Merck Manual. 16th ed. Rahway, NJ:Merck Research Laboratories, 812–821.
Gibbs RA, Hu CJ, Ho GE, Unkovich I. 1997. Regrowth of faecal coliforms and salmonellae in stored biosolids and soil amended with biosolids. Water Sci Technol 35:269–275.
Lewis DL. 1998. Microbes in the environment: challenges to exposure assessment. Presented at the Annual Meeting and Science Innovation Exposition of the American Association for the Advancement of Science, 12–17 February 1998. Philadelphia, PA.
Lewis DL, Garrison AW, Wommack KE, Whittemore A, Steudler P, Melillo J. 1999. Influence of environmental changes on degradation of chiral pollutants in soils. Nature. 401:898–901.
Lewis DL, Gattie DK. 2002. Pathogen risks from applying sewage sludge to land. Environ Sci Technol 36:286A–293A.
Lewis DL, Gattie DK, Novak M, Sanchez S, Pumphrey C. 2001. Interactions of pathogens and irritant chemicals in land-applied sewage sludge (biosolids). In: New Solutions, Vol 12, No 4 (Clapp R, Orlando L, eds). Amityville, NY:Baywood Publishing Co.
———. 2002. Interactions of pathogens and irritant chemicals in land-applied sewage sludges (biosolids). BMC Public Health 2:11.
Lewis DL, Shephard S, Gattie DK, Sanchez S, Novak M. 2000. Enhanced susceptibility to infection from exposure to gases emitted by sewage sludge: a case study. In: Proceedings of Biosolids Management in the 21st Century, 10–11 April 2000, College Park, MD. College Park, MD:Department of Civil & Environmental Engineering, University of Maryland, 168–174.
Liesivuori J, Kotimaa M, Laitinen S, Louhelainen K, Ponni J, Sarantila R, et al. 1994. Airborne endotoxin concentrations in different work conditions. Am J Ind Med 25(1):123–124.
Milton DK, Wypij D, Kriebel D, Walters MD, Hammond SK, Evans JS. 1996. Endotoxin exposure-response in a fiber-glass manufacturing facility. Am J Ind Med 29(1):3–13.
National Research Council. 2002. Biosolids Applied to Land: Advancing Standards and Practice. Washington, DC:National Academy Press.
NIOSH. 2002. Guidance for Controlling Potential Risks to Workers Exposed to Class B Biosolids. NIOSH Publication No. 2002-149. Cincinnati, OH:National Institute for Occupational Safety and Health. Available: http://www.cdc.gov/niosh/docs/2002-149/pdfs/2002-149.pdf [accessed 4 December 2003].
Notification and Federal Employee Antidiscrimination and Retaliation Act of 2002. 2002. Public Law 107-174.
Reimers RS, Oleszkiewicz JA, Shepherd SL, Bakeer RM, Fitzmorris KB. 2003. Advances in Alkaline Stabilization/Disinfection of Agricultural and Municipal Biosolids. Baltimore, MD:Water Environment Federation.
Rylander R. 1987. The role of endotoxin for reactions after exposure to cotton dust. Am J Ind Med 12(6):687–697.
Rylander R. 1995. Endotoxins in the environment. In: Lipopolysaccharides from Genes to Therapy (Levin J, Alving C, Munford R, Redl H, eds). New York:Wiley-Liss, 79–90.
Schiffman SS, Walker JM, Dalton P, Lorig TS, Raymer JH, Shustermann D, et al. 2000. Potential health effects of odor from animal operations, wastewater treatment, and recycling of byproducts. J Agromed 7(1):1–81.
Shields H. 2003. Sludge Victims. Fall 2002 Update. Alton, NH:Citizens for a Sludge-Free Land and New Hampshire Sierra Club.
Sigsgaard T, Malmros P, Nersting L, Petersen C. 1994. Respiratory disorders and atopy in Danish refuse workers. Am J Respir Crit Care Med 149(6):1407–1412.
Smid T, Heederick D, Houba R, Quanjer PH. 1994. Dust- and endotoxin-related acute lung function changes and work-related symptoms in workers in animal feed industry. Am J Ind Med 25(6):877–888.
U.S. EPA. 1993. 40 CFR Part 503. Fed Reg 58(32):9248–9415.
———. 2002. Land Application of Biosolids Status Report. 2002-S-000004. Washington, DC:U.S. Environmental Protection Agency, Office of Inspector General.
———. 2003a. Standards for the Use or Disposal of Sewage Sludge; Agency Response to the National Research Council Report on Biosolids Applied to Land and the Results of EPA’s Review of Existing Sewage Sludge Regulations. Fed Reg 68:17379–17395.
———. 2003b. Standards for the Use or Disposal of Sewage Sludge; Agency Response to the National Research Council Report on Biosolids Applied to Land and the Results of EPA's Review of Existing Sewage Sludge Regulations. Docket No. OW-2003-0006. Washington, DC:U.S. Environmental Protection Agency. Available http://cascade.epa.gov/RightSite/dk_public_home.htm [accessed 5 December 2003].
U.S. House of Representatives, Committee on Science. 2000a. EPA’s Sludge Rule: Closed Minds or Open Debate? No. 106-95. Washington, DC:U.S. Government Printing Office.
———. 2000b. Intolerance at EPA—Harming People, Harming Science? No. 106-103. Washington, DC:U.S. Government Printing Office.
Williams KL, ed. 2001. Pyrogen, endotoxin, and fever. In: Endotoxins: Pyrogens, LAL Testing, and Depyrogenation. 2nd ed. New York:Marcel Dekker, Inc., 12–24.
Yi ES. 2002. Hypersensitivity pneumonitis. Crit Rev Clin Lab Sci 39(6):581–629.

Applying processed sewage sludges (biosolids) to crop land, strip mines, public parks, and other areas has become common in the United States and elsewhere. This practice, in which several tons or more of processed municipal wastes are applied per acre annually, is regulated by the U.S. Environmental Protection Agency (EPA) under the 503 sludge rule (U.S. EPA 1993). The rule provides guidance for the beneficial reuse of municipal wastes and sets standards intended to protect public health and the environment from exposure to heavy metals, toxic chemicals, and pathogens. In recent years, land application has been increasingly scrutinized because of nuisance complaints and growing numbers of anecdotal reports of illnesses and deaths attributed to exposure to commercially processed sewage sludges.

Our laboratories investigated public complaints and concluded that irritant chemicals associated with volatile chemicals and dusts blowing from treated land (e.g., bacterial toxins, lime, organic amines) may cause nearby residents to be more susceptible to infections (Lewis et al. 2002, Lewis and Gattie 2002). We documented an outbreak of *Staphylococcus aureus* among individuals exposed to a Pennsylvania land-application site and attributed the infections to secondary exposure routes (animal-to-human or person-to-person). Overall, we questioned the efficacy of methods used to treat sewage sludges and determine pathogen levels; and, we recommended that new research focus on chemical-pathogen interactions, airborne contaminants (especially organic dusts), and risks posed to immunocompromised individuals and other sensitive populations (Lewis 1998; Lewis et al. 1999, ^2000^, 2001, 2002; Lewis and Gattie 2002).

The National Research Council (NRC 2002) echoed these same concerns, and the U.S. EPA intends to address some of the issues through additional research (U.S. EPA 2003a). In this paper we provide a more detailed overview of the risks that land application of sewage sludge poses to human health and how those risks can be better managed.

## High-Level Disinfection

Current federal standards for pathogen reduction in sewage sludge are based on levels of indicator organisms, such as *Escherichia coli* and *Salmonella*. Class A sludges have no detectible pathogens, whereas low levels of indicator pathogens are permitted in class B sludges. Sludges contain a wide variety of bacteria, viruses, protozoa, fungi, and parasitic worms, including some species that are more difficult to kill than the indicator organisms.

Table 1 shows levels of disinfection required to destroy different groups of pathogens found in sewage sludges. Low-level disinfection reduces numbers of vegetative bacteria (e.g., *E. coli*, *Salmonella*) and enveloped viruses [e.g., hepatitis B, human immunodeficiency virus (HIV), influenza viruses]. More resistant organisms require intermediate-level disinfection. These include mycobacteria (e.g., *Mycobacterium tuberculosis*), protozoa (e.g., *Cryptosporidium*, *Giardia*), parasitic worms (e.g., *Ascaris*, *Toxocara*) and fungi (e.g., *Candida*). Intermediate to high-level disinfection is required to kill some of the most important pathogens found in sludges, including small, nonenveloped viruses (e.g., *Norovirus*) and bacterial endospores (e.g., *Clostridium perfringens*).

Nonenveloped viruses comprise an important group of pathogens that require a higher level of disinfection than the indicator organisms recommended in the 503 rule (U.S. EPA 1993). Rotaviruses, for example, cause 30-40% of acute diarrhea that requires infants to be hospitalized, and *Norovirus* (Norwalk-like viruses) is responsible for 40% of the cases of nonbacterial diarrhea in children and adults (Berkow and Fletcher 1992). Other important infectious agents in this group include hepatitis A, hepatitis E, encephalomyocarditis virus, polioviruses, coxsacki viruses, reoviruses, rhinoviruses, astroviruses, caliciviruses, echoviruses, parvoviruses, and aphthovirus. Many of these viruses pose a particular threat to infants, the elderly, and individuals with chronic diseases.

The National Institute for Occupational Safety and Health (NIOSH 2002) recently concluded that Class B biosolids likely contain infectious levels of bacteria, viruses, protozoa, and helminths and recommended that workers use protective gear and take basic infection control precautions when handling the material. In issuing these guidelines, NIOSH acknowledged that current methods for processing Class B sewage sludges may fail to achieve even low-level disinfection.

Also recognizing that freshly processed Class B sludges may pose a significant risk of infection under certain conditions, the U.S. EPA included protective measures in the 503 rule (U.S. EPA 1993), such as temporarily restricting public access to Class B land-application sites with warning signs and fences. The U.S. EPA, however, failed to consider some potentially important exposure factors; for example, dusts from treated fields could expose surrounding communities, and certain chemicals in sludge may increase risks of infections. Moreover, stockpiling sludge and spreading it without incorporating it into soil are commonplace. In practice, the 503 rule is ineffective in preventing public exposure.

Based on the types of pathogens present in municipal wastes, sewage sludges should be treated with high-level disinfection. To meet this standard, treatment methods should demonstrate the ability to kill even the most resistant organisms, including nonenveloped viruses and bacterial spores. Because all federal and state requirements are based on less-resistant indicator organisms, it is not known whether current methods, including aerobic and anaerobic digestion, heat treatment, lime stabilization, and composting, could achieve high-level disinfection.

## Pathogen Regrowth

Although high-level disinfection would afford greater protection for both workers and the public from pathogens in freshly processed sewage sludge, the public can also be exposed to pathogens that proliferate after the sludge is applied (Gibbs et al. 1997). Viruses do not replicate outside their hosts; therefore, pathogen regrowth is mainly of concern with bacteria and fungi. Consequently, while viruses and other pathogens die off in the field, some pathogens may rebound. Also, new pathogens are introduced when sludge is mixed with soil and comes in contact with insects, birds, mammals, and other environmental sources of pathogens.

The potential for pathogen regrowth is the downside to sewage sludge being rich in nutrients that promote the growth of bacteria and fungi. The problem is similar to food poisoning with perishable foods, such as egg products. Eggs, like raw sewage, are often contaminated with *Salmonella*. With a little cooking, however, egg-containing products are safe for human consumption. Nevertheless, unless these foods are desiccated or refrigerated, other pathogens, such as *S. aureus,* multiply in them. The source of *S*. *aureus* in spoiled food is not the eggs, however, but normal skin microflora from the hands of people who prepare or handle the food.

Although sewage sludge is not a food product, the principle is the same. Sludge is rich in proteins and other nitrogen-rich organic compounds that promote the growth of *S*. *aureus* and other bacteria. These organisms multiply as sludges decompose in soil, and can present a risk of infection when traces of sludge enter skin abrasions or when the dusts contact mucous membranes or are inhaled. The risk is particularly high when sewage sludge contacts tissues injured by chemical irritants, burns, cuts, or abrasions. People with chronic diseases and compromised immune systems are especially at risk.

Also, as is the case with food products, sewage sludge that is heated or otherwise treated to kill pathogens is still subject to pathogen regrowth. In fact, because most of the competing microorganisms are eliminated, it is even more conducive to pathogen regrowth. Leaving pathogens in sewage sludge, however, is not the solution.

Unfortunately, pathogen regrowth is an inherent problem with all sludges rich in proteins, amino acids, and other forms of organic nitrogen and sulfur--regardless of how they are processed. Once the materials are applied and become wet, they are colonized by bacteria and fungi; the materials then decompose and emit noxious odors in the form of organic amines, organic sulfides, and other small-molecular-weight compounds.

Offensive odors that form as sludge biologically decomposes in the field indicate pathogen regrowth because they are produced as bacteria break down proteins and other organic compounds containing nitrogen and sulfur. Most treatment methods produce sludges that are only temporarily stable; that is, the sludges produce noxious odors from biological decomposition after they are applied in the field.

One commercial process achieves long-term stability by chemically reacting sludge under heat and pressure at high pH to drive off organic nitrogen as ammonia (Reimers et al. 2003). With this process, the combination of gaseous ammonia, high temperature, and pressure effectively eliminates a wide range of pathogens. The final wet product, which is odorless and has a high pH, is used to amend acidic soils. Because the nitrogen content is driven off, however, the product lacks nutrient value.

## Bacterial Toxins

Most bacteria found in sewage sludge produce either endotoxins or exotoxins, both of which can cause severe illness or death. As sludges decompose, toxins can leach into groundwater, enter surface water runoff, and be carried away in airborne dusts. Considering that tons of decomposing sewage sludge per acre are often applied to hundreds or thousands of acres many times a year, land-application sites have a potential for producing and exporting large quantities of toxins.

Exotoxins--proteins and peptides secreted into the surrounding environment by growing cells--are produced by both gram-negative and gram-positive bacteria. They are usually the most toxic of the two general types of bacterial toxins. Because they can retain their toxicity at extremely high dilutions, some exotoxins, including staphylococcal enterotoxins and shigatoxin, are used as biological warfare agents.

Although exotoxins are generally heat labile and could therefore be destroyed by heat-treatment processes for sewage sludges, treated sludges are still likely to become contaminated with *E. coli*, *Pseudomonas auruginosa,* and other exotoxin-producing bacteria in the field. Severe gastrointestinal illnesses reported by individuals using private wells near land-application sites may have been caused by exotoxins leaching into groundwater.

The same property that makes *S. aureus* a common cause of food poisoning--its ubiquitous presence--may also make it one of the more common pathogens to proliferate in sewage sludges after they are applied to land. The organism produces an exotoxin that is not destroyed by cooking. Symptoms caused by *S. aureus* food poisoning (e.g., nausea, cramps, vomiting) are due to the presence of this toxin. Land-application sites with high levels of *S. aureus* could contaminate air and water with potentially harmful levels of both the organism and its toxin.

Endotoxins, on the other hand, are lipopolysaccharide complexes in the cell walls of gram-negative bacteria only. They are associated with proteins and other components of the cell walls and are released when the bacteria die and cell walls break apart (Rylander 1995). Endotoxins are produced in large quantities when wastes colonized with gram-negative bacteria are treated (Sigsgaard et al. 1994). They would also be produced as gram-negative bacteria growing in nutrient-rich sludges die off in the field.

Unlike most exotoxins, endotoxins are heat stable even upon autoclaving (Baines 2000). They can, however, be inactivated with dry heat at > 200^o^C for 1 hr (Williams 2001). Traces of endotoxins in food and water can cause headaches, fever, fatigue, and severe gastrointestinal symptoms; however, their primary target is the lungs. In addition to the former symptoms, inhaling endotoxin-contaminated dusts can cause acute airflow obstruction, shock, and even death. Chronic respiratory effects can also develop [American Conference of Government Industrial Hygienists (ACGIH) 1999].

Allergic and nonallergic reactions caused by airborne endotoxins have been documented with exposures of 45-150 endotoxin units (EU)/m^3^ and 300-400 EU/m^3^ (Milton et al. 1996; Smid et al. 1994). Nearby residents exposed to dusts from land-application sites report many of the same symptoms of endotoxin poisoning that have been documented among sewage treatment plant workers. These include flu-like symptoms, nausea, vomiting, diarrhea, headaches, and difficulty breathing (Lewis et al. 2002). Rylander (1987) proposed occupational exposure limits to endotoxin-contaminated cotton dusts. Based on average air concentrations over an 8- to 10-hr workday, he suggested limits ranging from 200 EU/m^3^ to prevent airway inflammation to 20,000 EU/m^3^ to avoid toxic pneumonitis. The exposure levels of endotoxin-contaminated aerosols with sewage treatment plant workers have ranged from 80 to 4,100 EU/m^3^ (Liesvuori et al. 1994). The toxins, however, have a greater effect on people with immune systems compromised by injury or illness (Baines 2000).

## Chemical-Pathogen Interactions

Although many chemical contaminants found in processed sewage sludges may potentially interact with pathogens to cause, facilitate, or exacerbate the disease process through allegeric and nonallergic mechanisms, microbial by-products formed during the processing and decomposition of sewage sludge probably account for most of the acute health effects. Complaints among residents living near land-application sites are primarily respiratory related and are consistent with hypersensitivity reactions, including fever, cough, difficulty in breathing, nausea, and vomiting.

Numerous diseases involving immunologically mediated hypersensitivity reactions have been documented among workers exposed to organic dusts containing microbial products. Yi (2002) listed 27 diseases, each categorized according to the source of the dusts and the specific microorganisms identified as the primary cause of hypersensitivity. Sources include, for example, dusts from molded hay, mushroom compost contaminated with fungi and actinomycetes, *Streptomyces*-contaminated fertilizers, *Caphaloporium*-contaminated sewage, and wood contaminated with *Bacillus subtilis*.

Byssinosis, perhaps the most studied of these diseases, is attributed to traces of endotoxins from the breakdown of *E. coli* and other gram-negative bacteria on raw cotton fibers. Similarly, illnesses have been documented among wastewater treatment plant workers exposed to endotoxins in aerosols (Rylander 1987). Usually, the disease affects only a small percentage of sensitive workers.

Compared with waste treatment plant aerosols, however, endotoxin levels are probably much higher in sewage sludge dusts, which contain large numbers of predominantly gram-negative bacteria killed during treatment processes and after land application. Consequently, the frequency and severity of hypersensitivity among groups exposed to sewage sludge dusts may be much greater compared with exposure to other organic dusts.

Respiratory-related hypersensitivity is generally reversible when affected individuals are removed from the source of exposure and treated with high doses of corticosteroids. Corticosteroids used to treat the underlying inflammation, however, seriously impair the immune system. In the case of sewage sludge, this would render hypersensitive individuals highly susceptible to infection from the low levels of viruses, bacteria, fungi, and other pathogens in processed sludges.

Treating residents near land-application sites who experience hypersensitivity to processed sewage sludges, therefore, is both costly and risky. It would involve relocating affected individuals to another area, making certain that any potentially serious infections have been eliminated or controlled with antibiotics, then administering high doses of corticosteroids and closely monitoring for any new infections.

## Exposure Studies

A conference on health effects of odors sponsored by Duke University and the U.S. EPA concluded that gases and volatile emissions from waste products including processed sewage sludge may cause adverse health effects (Schiffman et al. 2000). While acknowledging the complexity of the problem, the participants recommended undertaking controlled studies of the odorous emissions. In responding to the NRC recommendations (NRC 2002), the U.S. EPA committed to measuring field concentrations of selected volatile and gaseous compounds at selected sites (U.S. EPA 2003a).

Land-applied sewage sludge can emit numerous volatile chemicals and gases that may act alone or in combination with one another to produce the kinds of symptoms reported by people living near biosolids-recycling operations. The composition of air contaminants emitted by any land-application site undoubtedly varies widely over space and time, as do the susceptibilities of individuals to the effects of these emissions. Consequently, it is unlikely that such research will adequately establish which components and combinations of components can potentially cause adverse health effects and under what conditions.

We propose an alternative approach with a more modest goal aimed at determining the extent to which emissions must be diluted to eliminate malodor complaints and irritant effects (e.g., burning eyes, coughing, breathing difficulties). Based on meteorologic data from local weather stations and publicly available topographic data, the dilution of air contaminants over areas surrounding land-application sites can be readily determined with an air-dispersion model (Lewis et al. 2002).

Meteorologic data should be collected over an extended period of time (e.g., 6-8 weeks) during the maximum potential exposure, for example, when land application is in progress, temperatures are high, and sufficient rainfall has occurred to support high levels of microbial activity. This approach does not require measuring specific pollutants, and a number of land-application sites could be studied with a reasonable level of resources.

In addition to collecting meteorologic data, local census data would be used in this type of study to randomly select two groups of residents of similar demographic compositions: one close to the land-application site (< 1 km) and one farther away (3-5 km). Individuals in each group would provide information on medical histories and keep daily records of the selected symptoms during the period when meteorologic data are collected. Using a similar approach, we found that residents living closer to land-application sites were more severely affected than those living farther away (Lewis et al. 2002).

Quantitatively, what is needed is a simple numerical index that captures the most important variables determining whether symptoms develop. The amount by which volatile emissions are diluted when they reach a residence and the number of times the dilution drops below a certain level may be sufficient for predicting whether odor and health-related complaints are likely to develop.

For example, an exposure index could be calculated based on *a*) the number of exposures in which levels of volatile chemicals at a residence are ≥ 10% of the levels over the sludged field and *b*) the average percent dilution for these exposures. This approach is illustrated by Equation 1, where *I_v_* is the exposure index for volatile emissions, *n* is the number of exposures in which levels of gases and volatile chemicals at a residence were ≥ 10% of the levels over the sludged field, and is the average dilution (percent) for all exposures ≥ 10% of the levels over the field.

I_v_ = *n*_•_ [1]

Once exposure indices and frequencies of symptoms are collected for a number of land-application sites, a representative dilution level required to eliminate odor complaints and acute adverse health effects can be determined. This index provides a quantitative measure of whether a land-application site is likely to cause odor complaints and acute adverse health effects at a particular location or distance from the site. Such information could be used to evaluate the effectiveness of treatment methods and land management practices.

## Public Concerns

To assess public concerns over the safety of current land-application practices, we distributed questionnaires to 150 individuals concerned about land-application of sewage sludges (Table 2). The group included farmers, residents complaining of adverse health effects, community leaders, and environmentalists. Based on the responses of 87 respondents from 15 states, a majority of respondents (51.7%) desired a total ban on land application of sewage sludges, while 35.6% believed that land application should just be suspended until proven safe. Most respondents (74.7%) lived near land-applications sites and most (67.5%) reported that they had been personally affected by the practice. Overwhelming malodor, vector attraction (flies, mosquitoes), and adverse health effects (e.g., difficulty breathing, chronic sinusitis) were the primary adverse effects reported by individuals living near the sites.

The need for additional research was strongly supported. Respondents, however, expressed little trust in federal and state environmental agencies to provide a reliable scientific evaluation of potential public health and environmental effects. More confidence was expressed if assessments were done by public health agencies, such as the Centers for Disease Control and Prevention (CDC) and the National Institutes of Health (NIH). Although few respondents (16.1%) reported engaging in environmental activism, the group rated environmentalist organizations as being the most trustworthy to assess the safety of land-application practices.

Overall, the survey results indicate that most people concerned about sewage sludge do not believe land application is inherently unsafe but object to the practice because they lack confidence in scientific studies funded by government and industry groups defending the status quo. By contrast, survey respondents indicated a greater level of confidence in studies of land-application practices if done by the CDC or the NIH. It appears, therefore, that overcoming opposition through additional scientific research will require strong involvement with respected public health organizations. It will also require supportive findings from researchers independent of the federal agencies and trade organizations that have historically overseen the development and marketing of land-application practices.

## Discussion

***Politics of land application.*** Since the 503 sludge rule (U.S. EPA 1993) was promulgated in 1993, the U.S. EPA, the U.S. Department of Agriculture, and the industry and its trade associations have vigorously defended the rule as fully protective of public health and the environment. The primary basis has been a lack of documented cases of illnesses and the results of research supported with congressional funds earmarked for promoting land application as safe and beneficial (U.S. EPA 2002).

In 2000, the Committee on Science in the U.S. House of Representatives held hearings into allegations that the U.S. EPA retaliates against scientists and private citizens who report adverse environmental and health effects associated with sewage sludge (U.S. House of Representatives 2000a, 2000b).

During the hearings, the U.S. EPA Office of the Inspector General released a report confirming that concerns were widespread among U.S. EPA scientists who reviewed the 503 rule (U.S. EPA 2002). The assistant administrator refused to approve the rule without a major commitment from the Office of Water to support additional in-house research within the Office of Research and Development. The Inspector General noted that this research was never carried out.

The U.S. EPA responded to the congressional hearings by calling for a study by the National Research Council; Congress debated, and overwhelmingly passed, the No Fear Act (Notification and Federal Employee Antidiscrimination and Retaliation Act of 2002). The act, which was aimed at better protecting employees against retaliation, was signed by President Bush last year. The NRC published its findings and recommendations in July 2002 (NRC 2002), and the U.S. EPA addressed them in a research strategy in the *Federal Register* earlier this year (U.S. EPA 2003a). The U.S. EPA's final response to the NRC report is due to be released in January 2004. In the meantime, the U.S. EPA Office of Water has provided a docket for public comments (U.S. EPA 2003b).

Public comments to the Office of Water docket have largely mirrored those in the survey we report in this article. There is an overall lack of confidence in the the U.S. EPA's willingness to conduct or support objective research in this area. As evidence, the Sierra Club (San Francisco, CA) and others pointed to the fact that the Office of Water intends to address the NRC recommendations extramurally by funding the same researchers it has historically supported with congressional appropriations for promoting the safety of biosolids. The NRC study (NRC 2002), therefore, had its beginning and ending rooted in controversy over the U.S. EPA using congressional appropriations to support federal policies on the beneficial reuse of sewage sludge and to oppose scientists and private citizens who question them.

***Trends in land application.*** With increasing numbers of residents who live near sludged fields reporting respiratory and gastrointestinal illnesses (Shields 2003), many local governments have banned land application of Class B sewage sludges. However, we found that some Class A sludges generate the same complaints and concluded that going to Class A products will not resolve the pathogens issue (Lewis and Gattie 2002). The reason for this is that infections appear to be primarily opportunistic, following irritation of the skin, mucous membranes, and respiratory tract by chemical components.

Consequently, an important aspect of preventing infections lies in reducing levels of microbial toxins and other chemicals that cause inflammation as well as other responses that predispose individuals to infection. As such, the infections arise from many sources, both community and environmental. The problem with Class A sludges is probably primarily endotoxin related. This is because gram-negative bacteria comprise much of the biomass and because most conditions used to kill bacteria in treatment processes are insufficient to break down endotoxins. The Class A standard, therefore, while reducing the risks of acquiring infections directly from processed sludge, could increase risks of infections from other environmental and community sources.

**Table 1:** Disinfection levels required to kill pathogens in sewage sludges.^*a*^

Group	Disinfection level required
Bacterial endospores (e.g., *Bacillus anthracis*)	High
Nonenveloped viruses (e.g., *Norovirus, Coxsackie, Rotavirus*)	Intermediate/high
Helminths (e.g., *Ascaris, Toxocara*)	Intermediate
Protozoa (e.g., *Cryptosporidium, Giardia*)	Intermediate
Mycobacteria (e.g., *M. tuberculosis*)	Intermediate
Fungi (e.g., *Candida*)	Low/intermediate
Vegetative bacteria (e.g., *Staphylococcus, Salmonella*)	Low
Enveloped viruses (e.g., hepatitis B, HIV, influenza)	Low
Data from the Association for the Advancement of Medical Instrumentation (AAMI 1994). ^*a*^Disinfection levels are based on susceptibilities to liquid chemical germicides; groups increase similarly in resistance to heat, with enveloped viruses being the most sensitive and bacterial endospores the most resistant.	

**Table 2:** Summary of survey results from 87 respondents indicating their level of public concern about land application practices.

Topic	Question	Choices	Percent or mean ± SD (*n*)
Background information^*a*^	Why are you interested in the issue of land-applied sewage sludges?				Live or lived near land application site	74.7% (87)
	Work as a farmer/grower	4.6% (87)
	Engaged in environmental activism	16.1% (87)
	Other	14.9% (87)
	Have you ever been personally affected by land application of sewage sludges?	Yes	67.5% (77)
	How do you think land application of sewage sludges should be handled?	Current practices are safe;no new restrictions are needed	0% (87)
		All land application should be completely banned	51.7% (87)
		Only certain land application practices should be banned	8.0% (87)
		All land application should be suspended until proven safe	35.6% (87)
		Land application should be continued with certain new restrictions	2.3% (87)
		Other	5.7% (87)
Level of concern^*b*^	On a scale of 0 to 10 (0 = no concern; 10 = highest level of concern), indicate your level of concern regarding the following issues	Microorganisms that may cause infection	9.7 ± 0.9 (83)
		Chemicals, metals and microorganism that may cause cancer	9.6 ± 1.0 (84)
		Odor-causing emissions	8.9 ± 1.9 (84)
		Bacterial toxins	9.7 ± 0.8 (82)
		Property value	8.6 ± 2.3 (82)
		Other	9.7 ± 0.7 (25)
	Kinds of contamination from sludges that cause the most concern (0 = no concern; 10 = highest level of concern)	Contamination of food supply	9.3 ± 1.4 (83)
		Contamination of water	9.9 ± 0.7 (84)
		Contamination of soil	9.8 ± 0.5 (84)
		Contamination of air	9.6 ± 1.0 (84)
		Other	9.8 ± 0.5 (26)
Level of trust^*b*^	Using a scale of 0 to 10 (0 = no trust; 10 = highest level of trust) indicate your level of trust in organizations dealing with land application of sewage sludges	Congress	2.0 ± 2.3 (81)
		U.S. EPA	1.3 ± 2.4 (82)
		U.S. Department of Agriculture	1.7 ± 2.5 (80)
		State agencies	1.2 ± 2.1 (81)
		Local governments (city/county)	2.4 ± 3.1 (81)
		Environmental organizations	7.0 ± 2.8 (81)
		Trade groups (e.g., WEF, NEBRA)	0.8 ± 2.1 (75)
		National Biosolids Partnership	0.5 ± 1.7 (72)
		Industry	0.5 ± 1.5 (80)
		Other^*c*^ (e.g., departments of health, independent scientists)	5.3 ± 4.4 (19)
Need for additional research^*b*^	On a scale of 0 to 10 (0 = don’t feel that more research is needed; 10 = feel very strongly that more research is needed), indicate how strongly you feel that more scientific research is needed before we will know whether land applying sewage sludges is safe for public health and the environment
	On a scale of 0 to 10 (0 = no trust; 10 = highest level of trust), indicate your level of trust in the work of scientists supported by various organizations dealing with land application of sewage sludges	National Science Foundation/National Institutes of Health	5.1 ± 3.3 (72)
		Trade groups (WEF/WERF)	1.4 ± 2.2 (72)
		U.S. EPA Office of Water	1.4 ± 2.4 (77)
		U.S. EPA Office of Research and Development	2.8 ± 3.1 (75)
		U.S. Department of Agriculture	1.8 ± 2.6 (76)
		Centers for Disease Control and Prevention	4.9 ± 3.4 (75)
		Industry	0.5 ± 1.4 (76)
		State agencies	1.2 ± 2.1 (78)
		Other^*c*^ (e.g., universities, independent scientists, environmental groups)	5.9 ± 4.7 (19)
Abbreviations: NEBRA, New England Biosolids and Residuals Association; WEF, Water Environment Federation; WERF, Water Environment Research Federation. Responses not following survey instructions were omitted. ^***a***^Based on yes/no responses from all 87 respondents; values shown are percent ± SD (*n*). ^***b***^Based on a 0–10 scale, with averages determined from the actual number of responses to each category; values shown are mean ± SD (*n*). ^***c***^Categories given by respondents.			

One outcome of local bans is that land application of sewage sludge is being forced out of areas where residents have the political and economic resources to oppose the practice and into economically depressed areas. Whether this is intentional or not, sewage sludge is being dumped more and more into those communities least able to have their complaints heard, and where residents are least capable of relocating or obtaining medical treatment.

The changing demographics of land application of sewage sludge, therefore, need to be studied. First, census data should be used to assess the socioeconomic makeup of communities living near land-application sites (< 1 km away). Steps then need to be taken to ensure that land-application practices do not disproportionately impact low-income and minority communities.

## Recommendations

We recommend that the U.S. EPA undertake and complete five top-priority measures by January 2006 to address the immediate adverse health and environmental effects associated with land-applied sewage sludges:

- Develop a universal high-level disinfection standard to replace the current Class A/Class B standards, and require industry to provide efficacy data showing treatment methods meet this standard for sporocidal, fungicidal, bactericidal, tuberculocidal, virucidal, anti-protozoal, and anti-parasitic activity
- Develop treatment methods and land management practices for reducing airborne endotoxin levels associated with processed sewage sludges and set limits for public exposure. (We recommend that maximum levels be set at 0.1 times the limit recommended for 10-hr occupational exposure for preventing airway inflammation, which would be 20 EU/m^3^.)
- Conduct a national assessment of groundwater contamination from pathogens, microbial (bacterial, fungal) toxins, organic chemicals, and metals at land-application sites
- Require that industry ensure that land-application practices do not disproportionately target low-income and minority subpopulations in rural communities
- Work with the National Biosolids Partnership (Association of Metropolitan Sewerage Agencies, U.S. EPA, and the Water Environment Federation) to develop and enforce a policy supporting open competition for research funding and prohibiting discrimination and retaliation against individuals raising concerns over adverse environmental and health effects from land-applied sewage sludges.
